# Utilization of a Fluorescent Marker in a Simulated Biological Fluid Spill Drill Within a Position Emission Tomography Center

**DOI:** 10.7759/cureus.31719

**Published:** 2022-11-21

**Authors:** Koon L Chia, Michael Tong, Lycia L Teo, Bertrand W Ang, Shao J Ong

**Affiliations:** 1 Radiology, National University Hospital, Singapore, SGP; 2 Psychiatry, Ng Teng Fong General Hospital, Singapore, SGP; 3 Radiology, National University of Singapore, Singapore, SGP

**Keywords:** skills and simulation training, radiation spill, surface contamination, clinical simulation, pet ct

## Abstract

The management of uncontained spillage of radioactive material in nuclear medicine healthcare facilities is documented in their standard operating procedures (SOPs). These are supplemented by periodic training drills for staff to practice the appropriate responses and decontamination techniques. We report on the use of Glo Germ (GloGerm Co., Moab, UT, USA), a commercially available abiotic powder that fluoresces under black light, as a visual aid in these spill simulations. Glo Germ was used in a spill drill scenario within the controlled area in the nuclear medicine department. This provided immediate visual feedback for the staff involved in the simulations as well as the supervision observers. We anticipate that the use of such aids during training will enhance confidence and proficiency in managing and decontaminating radiation spills. It will also serve to flag potential gaps in decontamination protocols and allow for the refinement of SOPs.

## Introduction

The nuclear medicine department is a vital service provider that uses unsealed radionuclide sources for both diagnostic imaging and therapeutic strategies for the treatment of patients. The use of unsealed sources is accompanied by the potential hazards of spills and consequent contamination. Radiation is undetectable by the human senses. As such, in the event of a spill, those in the vicinity are at risk of significant radiation exposure without even being aware of it.

Standard operating procedures (SOPs) are necessitated by both international guidance and local rules for a facility’s proper response to managing radiation spills [1]. Mandatory training and periodic simulations supplement the SOPs by allowing staff of all levels of experience to practice their response to manage and contain these unsealed sources. Routinely, these simulations use nonviscous, nontoxic, nonvolatile, and nonfluorescent substances such as tap water or saline instead of live sources during spill drills in line with the As Low As Reasonably Achievable (ALARA) principle.

The five stages of adult learning are dissonance, refinement, organization, feedback, and consolidation. Of these stages, feedback has been highlighted to be the most important because it is when the learner is aware of their current competency level and decides whether remedial action is needed [2].

While running scenarios, it is important to maximize the visual impact of the simulations to avoid participants becoming complacent and assuming proficiency. The use of typical substances such as water or saline does not provide any strong visual feedback to the drill participants on the effectiveness of their isolation and decontamination techniques.

To address this limitation, we innovated the use of a fluorescent marker as a training tool to simulate the transfer of radioactive contaminants by direct contact between clean and contaminated surfaces during spill drills. In professions such as infection control and food preparation, fluorescent markers are widely used in the routine education of handwashing and aseptic procedures [3]. We propose its use in nuclear medicine spill drills as a simple, safe, and cost-effective method to visualize simulated contamination.

## Technical report

Glo Germ (GloGerm Co., Moab, UT, USA) is an abiotic fluorescent substance that is minimally visible under regular lighting but glows under ultraviolet (UV) illumination. It is available in powder, gel, liquid, and aerosol forms. In our simulation training, we utilized Glo Germ in its powder form.

At our institution, spill drills are periodically carried out in controlled areas in the nuclear medicine department. Our standard scenario involves a patient injected with 18F-fluorodeoxyglucose (18F-FDG) who then uncontrollably voided onto the floor in the sub-wait area. To simulate the urine, 200 mL of water mixed with half a teaspoon of Glo Germ powder was splashed onto the floor to simulate a contaminated area.

The simulations were carried out without participants being informed of the use of the fluorescent powder. At the end of the *expected* drill, ambient lighting was switched off and a 395-nM UV handheld torch was used to assess the spread and containment of the simulated contamination. The participants were then able to observe the spread of the powder and draw conclusions on how to improve their responses in an actual spill.

In the event of a spill, SOPs call for nuclear medicine-trained staff to visually demarcate the contaminated area and commence decontamination efforts. Simultaneously, facility staff (staff from nonspecialized hospital central services) are required to respond and assist to cordon off a wider zone around the presumed contaminated area.

**Figure 1:**
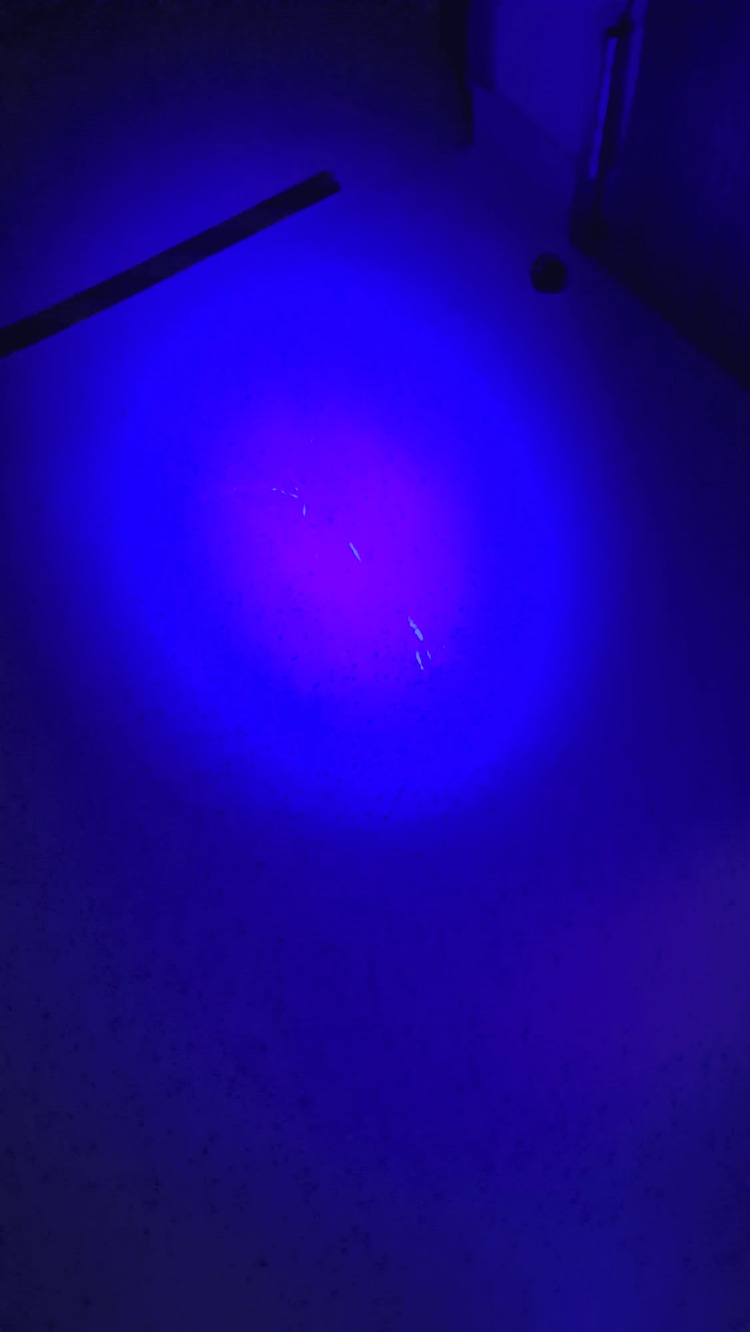
Residual streaking of Glo Germ powder visualized under a 395-nM ultraviolet handheld torch due to insufficient cleaning of the original contamination area.

Following the cleaning of the contaminated area as per SOPs, residual traces of powder in thin, streak-shaped patterns were found, demonstrating where contamination was not cleanly wiped off the floor (Figure 1). The clear visualization of the residual contamination provided good instant feedback on the cleaning method performed by the nuclear medicine-trained staff.

**Figure 2:**
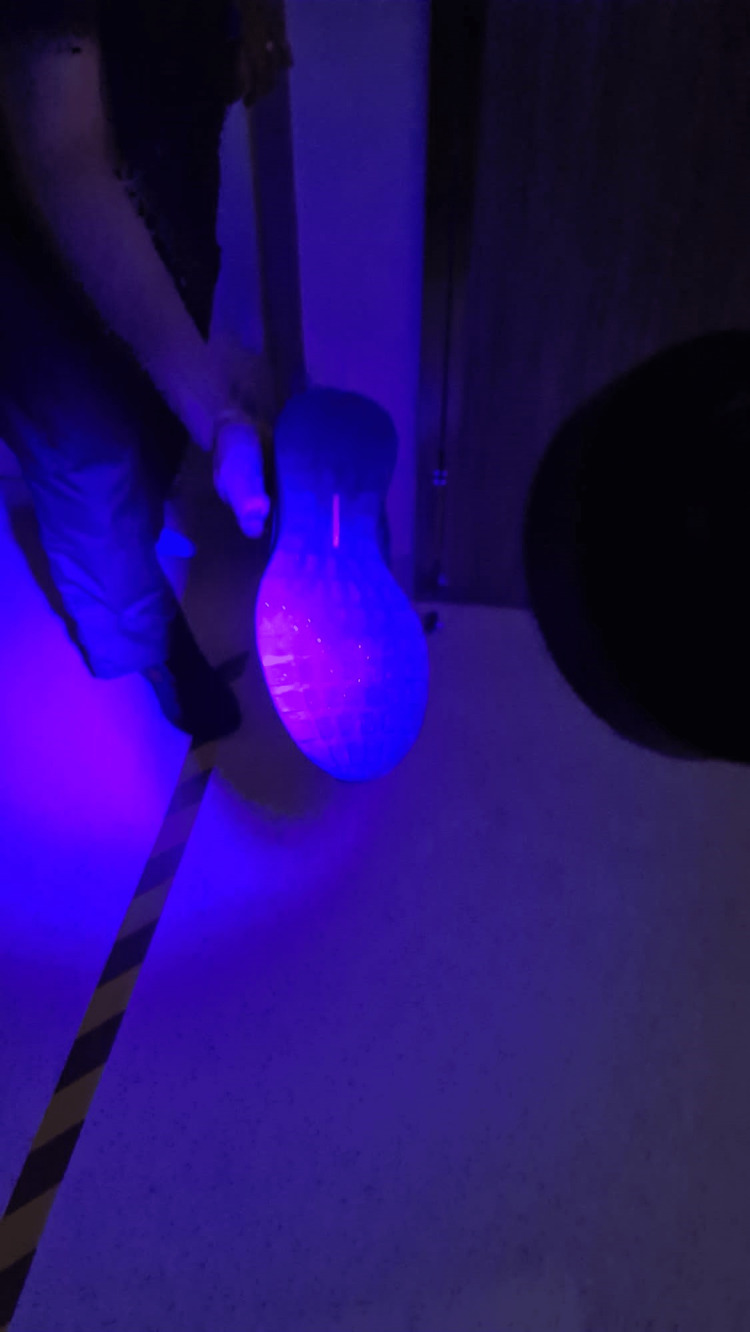
Cross-contamination of Glo Germ powder on the shoes of a facility member who passed through the sub-wait area to set up the exclusion zone.

In addition, a facility staff member (male) who passed through the sub-wait area to set up the exclusion zone was found to have residual contamination on his shoes (Figure 2). 

During the postsimulation debrief, all participating staff agreed that using Glo Germ significantly helped to highlight the potential contamination that could be transferred to themselves or their personal protective equipment (PPE).

The nuclear medicine staff practicing decontamination were able to receive immediate visual feedback and clearly understood how to improve their technique. A facility staff member (male) who was not required by SOPs to wear PPE such as shoe covers immediately realized his mistake in assuming that contamination was present only within the demarcated area. Supervisors and observers from nuclear medicine and facility services were able to assess the weaknesses and strengths of their existing SOPs and make necessary adjustments.

## Discussion

To the best of our knowledge, this is the first reported use of a fluorescent powder in nuclear medicine spill drills for training and education purposes. Other medical professions, such as anesthesia [4], have increasingly started making use of Glo Germ as a visual indicator during training and simulation. The use of Glo Germ in nuclear medicine is simply a lateral application, as unsealed radiation sources and biotic contaminants have very similar hazards.

Spill drill scenarios typically assume a limited area of contamination when an unsealed source is dropped from a height. Our staff is trained to demarcate a reasonably broad area around the point of impact or to include as much visible fluid as possible. Examining the wider spill area in the nuclear medicine department under UV light revealed that small droplets of the water-Glo Germ mixture were present up to a few meters away from the visible spatter pattern and the demarcated area. This is potentially where the facility staff member picked up the contamination onto his shoes. This highlights our concern that containment of the daylight visible spill substitutes is not sufficient to provide a good assessment of the overall decontamination proficiency. The significance of the hazard would depend on the radionuclide that was spilled, for example, short-lived, nonvolatile 99mTc-chloride versus β-emitting volatile 90Y microspheres.

In comparison to Glo Germ, the use of water as a radiation-free substitute was shown to provide minimal visual feedback. During training, a spill is typically assessed to be contained when no visible fluid can be observed; the presence of Glo Germ residue in our drills indicated otherwise. We emphasize that the use of Glo Germ is simply a training tool, as in a real radionuclide spill the use of a survey meter is still the gold standard to detect contamination.

We anticipate that Glo Germ can be possibly used to score or compare decontamination techniques, for example, wiping the contaminated area repeatedly in the same direction versus a circular motion. Spatter patterns of different types of spills can be studied and SOPs revised to mitigate changes in risk assessments.

## Conclusions

The uncontrolled spread of radiation contamination, and subsequent radiation exposure, is of paramount concern to both staff and the public in a nuclear medicine facility. It is imperative that staff members are well-equipped and prepared to deal with any unexpected spills as quickly and efficiently as possible to minimize radiation exposure to anyone in the vicinity.

The main benefit of using Glo Germ in our simulation training is to provide immediate visual feedback on decontamination techniques and to dispel the misconception that contamination is only present in the arbitrarily demarcated area. The simulation and associated spatter pattern of the simulated contaminant is more realistic and is an improvement on using a radiation-free substitute such as water or saline.

Future avenues in which Glo Germ could be utilized may include assessing the effectiveness of different decontamination techniques. The use of different Glo Germ formulations such as lotion and powder in water can aid in making simulations involving viscous and nonviscous contaminants (e.g., blood and urine) more realistic and visually impactful.

We opine that primary and secondary responders to a radiation spill will have better confidence and less anxiety in dealing with an unexpected contamination situation after undergoing simulation training. The use of Glo Germ as a visual feedback tool enhances its effectiveness and impact and allows radiation safety officers and management to intuitively refine SOPs.
